# Phase 3 Study Assessing Lot-to-Lot Consistency of Respiratory Syncytial Virus Prefusion Protein F3 Vaccine and Its Immune Response, Safety, and Reactogenicity When Co-administered With Quadrivalent Influenza Vaccine

**DOI:** 10.1093/infdis/jiae342

**Published:** 2024-07-05

**Authors:** Nnenna Chime, Bruno Anspach, Vishal Jain, Outi Laajalahti, Thierry Ollinger, Deborah Yaplee, Joon Hyung Kim

**Affiliations:** GSK, Rockville, Maryland; GSK, Rockville, Maryland; GSK, Rockville, Maryland; Finnish Vaccine Research, Seinäjoki, Finland; GSK, Wavre, Belgium; GSK, Rockville, Maryland; GSK, Rockville, Maryland

**Keywords:** influenza virus, quadrivalent influenza vaccine, respiratory syncytial virus, RSVPreF3 vaccine, vaccine co-administration

## Abstract

**Background:**

A single-dose investigational respiratory syncytial virus (RSV) vaccine, RSV prefusion protein F3 (RSVPreF3), was co-administered with a single-dose quadrivalent influenza vaccine (FLU-D-QIV) in a phase 3, randomized, controlled, multicenter study in healthy, nonpregnant women aged 18–49 years.

**Methods:**

The study was observer-blind to evaluate the lot-to-lot consistency of RSVPreF3, and single-blind to evaluate the immune response, safety, and reactogenicity of RSVPreF3 co-administered with FLU-D-QIV.

**Results:**

A total of 1415 participants were included in the per-protocol set. There was a robust immune response at day 31 across each of the 3 RSVPreF3 vaccine lots; adjusted geometric mean concentration ratios (95% confidence interval [CI]) were 1.01 (.91–1.12), 0.93 (.84–1.03), and 0.92 (.83–1.02) for RSV1/RSV2, RSV1/RSV3, and RSV2/RSV3, respectively. For FLU-D-QIV co-administered with RSVPreF3, versus FLU-D-QIV alone at day 31, noninferiority was satisfied for 3 of 4 strains assessed, with the lower limit of the 95% CI for geometric mean ratio >0.67.

**Conclusions:**

Immunogenic consistency was demonstrated for 3 separate lots of RSVPreF3. Immunogenic noninferiority was demonstrated when comparing FLU-D-QIV administered alone, versus co-administered with RSVPreF3, for 3 strains of FLU-D-QIV. Co-administration was well tolerated, and both vaccines had clinically acceptable safety and reactogenicity profiles.

**Clinical Trials Registration:**

NCT05045144; EudraCT 2021-000357-26.

## Teaser

This study demonstrated the lot-to-lot consistency of 3 lots of respiratory syncytial virus prefusion protein F3 (RSVPreF3) vaccine and highlighted that RSVPreF3 and FLU-D-QIV co-administration was immunogenically noninferior to the administration of FLU-D-QIV alone and was well tolerated.

Respiratory syncytial virus (RSV) exists as 2 antigenically distinct subgroups, RSV-A and RSV-B [1]. It is a highly contagious human pathogen that causes respiratory tract infections in people of all ages, although infants aged <2 years have the highest incidence of severe disease (eg, bronchiolitis and viral pneumonia), with peak incidence at age 1 month [1–4].

In temperate climates, RSV typically causes fall–winter epidemics, with a mid-winter peak [3, 5]. However, in tropical and subtropical regions, viral activity is more endemic and outbreaks are less temporally focused [6, 7]. Severe RSV disease often leads to hospitalization and may be life threatening [6–10]. The risk of severe RSV-induced lower respiratory tract disease (LRTD) is highest in infants aged <6 months; such LRTDs are the most common cause of hospitalization in this age group [8]. Moreover, an increased risk of severe RSV disease is associated with premature birth (gestation age ≤35 weeks) [3].

Since 1998, the humanized monoclonal antibody palivizumab has been used in infants at high risk of RSV infection, albeit to a limited extent due to cost issues and ambiguity around the most appropriate indications [9, 11]. In November 2022, the European Medicines Agency approved, as a single intramuscular dose, nirsevimab, a recombinant human immunoglobulin (Ig) G1κ monoclonal antibody, for the prevention of RSV LRTD in neonates and infants during their first RSV season [12, 13]. In July 2023, the US Food and Drug Administration (FDA) approved nirsevimab for the prevention of RSV LRTD in neonates and infants during their first RSV season, and in children aged ≤24 months who remain vulnerable to severe RSV disease through their second RSV season [13, 14]. An RSV prefusion F vaccine (RSVPreF [Abrysvo]; Pfizer Inc, New York, New York) was recently approved by the FDA for the active immunization of pregnant women at 32–36 weeks of gestational age for the prevention of LRTD and severe LRTD caused by RSV in infants from birth through 6 months of age [15, 16].

Pregnant women are at increased risk of serious consequences from influenza, which can lead to adverse pregnancy outcomes; therefore, in many countries, use of a seasonal inactivated influenza vaccine is recommended during pregnancy to protect against serious complications [17]. FLU-D-QIV, a quadrivalent vaccine against influenza (available as Fluarix Quadrivalent, Fluarix Tetra, α-RIX-Tetra, or Influsplit Tetra; GSK), is recommended for administration during the second or third trimester of pregnancy in many countries [17].

An investigational RSV vaccine, RSV prefusion protein F3 (RSVpreF3), was in late-stage development for administration to pregnant women, with the aims of transferring maternal antibodies and preventing RSV-associated LRTDs in infants. The investigational RSVPreF3 vaccine (not adjuvanted) was intended for administration in the second or third trimester of pregnancy, and evaluating any potential interference from co-administration with FLU-D-QIV vaccine was an important part of the development program. However, development of this vaccine was discontinued following a safety signal [18]. This study was conducted prior to the identification of the safety signal and was designed to evaluate the lot-to-lot consistency of the investigational RSVPreF3 maternal vaccine, and the immune response, safety, and reactogenicity of the RSVPreF3 maternal vaccine when co-administered with FLU-D-QIV.

## METHODS

Additional methodologic details are in the Supplementary Materials.

### Study Design and Participants

**Figure 1.**
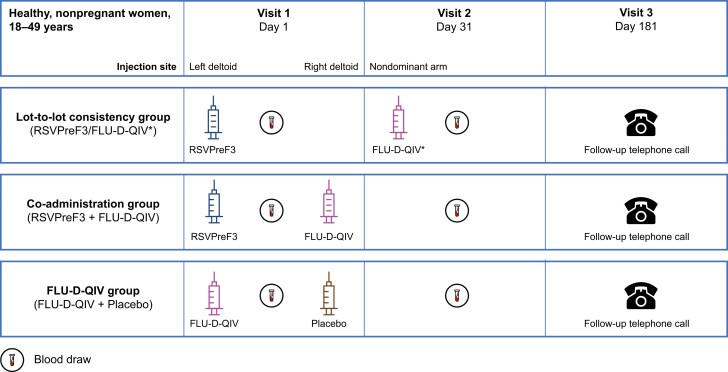
Study design. *Optional vaccination (not part of the experimental design) as part of standard of care. Abbreviations: FLU-D-QIV, quadrivalent influenza vaccine; RSVPreF3, respiratory syncytial virus prefusion protein F3 vaccine.

This was a phase 3, randomized, controlled, multinational, multicenter study (from 15 September 2021 to 6 June 2022) in healthy, nonpregnant women aged 18–49 years. The study was observer-blind (part A) to evaluate the lot-to-lot consistency of RSVPreF3 and single-blind (part B) to evaluate the immune response, safety, and reactogenicity of RSVPreF3 when co-administered with FLU-D-QIV (Figure 1).

**Figure 2.**
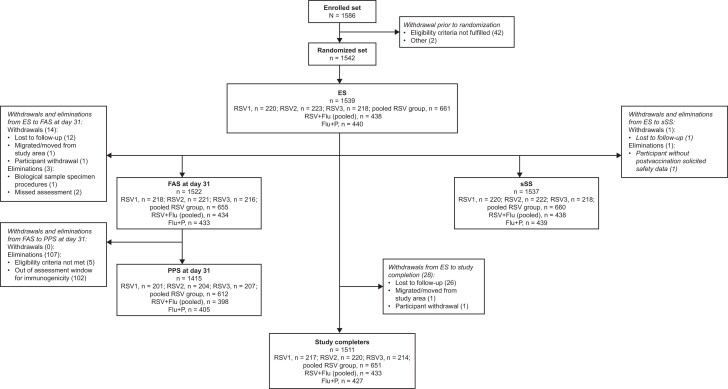
Study flow. Abbreviations: ES, exposed set; FAS, full analysis set; Flu, FLU-D-QIV quadrivalent influenza vaccine; P, placebo; PPS, per-protocol set; RSV, respiratory syncytial virus; RSV1, respiratory syncytial virus prefusion protein F3 (lot 1); RSV2, respiratory syncytial virus prefusion protein F3 (lot 2); RSV3, respiratory syncytial virus prefusion protein F3 (lot 3); sSS, solicited safety set.

In study part A, participants were randomized into 3 groups (RSV1, RSV2, and RSV3); each group received RSVPreF3 120 μg, from 1 of the 3 lots of the RSVPreF3 vaccine (Figure 2). Participants in the lot-to-lot consistency group were given the opportunity to receive standard of care, FLU-D-QIV at day 31. In study part B, participants received RSVPreF3 co-administered with FLU-D-QIV (RSV + Flu [pooled] group), or received FLU-D-QIV co-administered with placebo (Flu + P).

All study participants who received a study intervention were followed for safety and reactogenicity and were evaluated for solicited administration-site and systemic adverse events (AEs) occurring within 7 days of vaccination, unsolicited AEs occurring within 30 days of vaccination, and serious AEs (SAEs) and pregnancy outcomes throughout the study period (up to day 181).

### Eligibility Criteria

Study participants were healthy women aged 18–49 years at the time of the first study vaccine administration; who were able, in the opinion of the investigator, to comply with the requirements of the study protocol; and had no local condition precluding injection in the left and right deltoid muscles. Study participants of childbearing potential were enrolled if they had practiced adequate contraception for 1 month prior to study vaccine administration, had a negative pregnancy test on the day of study vaccine administration, and had agreed to continue adequate contraception for 1 month after completion of study vaccine administration.

### Randomization

Randomization used an automated, internet-based system (Source Data Base for Internet Randomization).

### Blinding

Data were collected in an observer-blind manner to evaluate the lot-to-lot consistency of RSVPreF3 vaccine, and in a single-blind manner to evaluate the immune response, safety, and reactogenicity of RSVPreF3 co-administered with FLU-D-QIV.

### Study Interventions

RSVPreF3 120 μg (0.5 mL) was administered as a single intramuscular dose in the left deltoid muscle (Supplementary Table 1). FLU-D-QIV contained 15 μg hemagglutinin per strain per dose and was administered as a single intramuscular dose in the right deltoid muscle, except for the dose at day 31, which was administered in the nondominant arm. The 4 constituent influenza strains in the FLU-D-QIV vaccine were A/Victoria/2570/2019 (H1N1), IVR-215; A/Tasmania/503/2020 (H3N2), IVR-221; B/Washington/02/2019; and B/Phuket/3073/2013. Normal saline solution was used as the placebo control and was administered as a single intramuscular dose in the right deltoid muscle.

### Study Objectives

The key immunogenicity objectives were to demonstrate the lot-to-lot consistency of 3 lots of the investigational RSVPreF3 vaccine, based on geometric mean concentration (GMC) ratios for RSVPreF3 IgG enzyme-linked immunosorbent assay (ELISA) titers at day 31 (postvaccination), and to demonstrate noninferiority for FLU-D-QIV vaccine, when co-administered with RSVPreF3 vaccine, compared with FLU-D-QIV alone, based on hemagglutination inhibition (HI) geometric mean titer (GMT) ratios for antibodies against 4 constituent influenza strains at day 31 postvaccination. The study was originally designed to assess noninferiority for all 4 strains; however, after clinical development of the maternal RSVPreF3 was discontinued due to a safety signal in another study [18], and with logistic challenges, the A/Victoria/2570/2019 (H1N1) strain was tested only as a tertiary objective.

Secondary, confirmatory immunogenicity objectives were to demonstrate noninferiority of RSVPreF3 vaccine co-administered with FLU-D-QIV, compared with RSVPreF3 alone, based on neutralizing GMT ratios against RSV-A at day 31 postvaccination; and demonstrate noninferiority of FLU-D-QIV co-administered with RSVPreF3, compared with FLU-D-QIV alone, based on seroconversion rates regarding HI antibody titers against 4 constituent influenza strains at day 31 postvaccination. Seroconversion rate was the proportion of participants with day 1 (prevaccination) serum anti-HI titer <1:10, and day 31 (postvaccination) serum anti-HI titer ≥1:40; or day 1 serum anti-HI titer ≥1:10 and ≥4-fold increase (post- vs prevaccination) at day 31. Seroprotection rate, a descriptive objective, was the proportion of participants with serum HI titer ≥1:40 at day 31. A hierarchical procedure was used for the assessment of confirmatory objectives (see Supplementary Materials for further details).

Primary safety objectives were to evaluate the safety and reactogenicity of RSVPreF3 alone (pooled RSV1 + RSV2 + RSV3 [pooled RSV] group), or co-administered with FLU-D-QIV, up to study end. Data were expressed as the percentage of participants in each group reporting solicited administration-site or systemic AEs within 7 days of vaccination, unsolicited AEs within 30 days of vaccination, or SAEs during the study period (day 1 to day 181).

### Immunogenicity Assays

Blood samples for humoral immune responses were collected from all participants prior to vaccination at visit 1 (day 1) and 1-month postvaccination (visit 2 [day 31]). Anti-RSVPreF3 binding IgG antibodies were measured using ELISA using a cut-off of 25 ELISA units (EU)/mL. RSV-A and RSV-B neutralizing titers were determined using an in-house RSV serum neutralization assay. The assay cut-off was 18 estimated dilution 60 (ED_60_) for RSV-A and 30 ED_60_ for RSV-B. HI antibody titers were determined using the method derived from the World Health Organization (WHO) Manual on Animal Influenza Diagnosis and Surveillance, WHO/CDS/CSR/NCS/2002 [19]. Additional information on assays is presented in the Supplementary Materials.

### Safety and Reactogenicity

Electronic diaries were used to collect solicited AE data up to and including day 7. A standardized electronic case report form (eCRF) was used to collect unsolicited AE data up to and including day 30. SAE data from day 31 until study end were collected through questioning at concluding phone contacts and were reported in the eCRF, as appropriate.

Safety endpoints, including solicited AEs, unsolicited AEs, SAEs, and AEs leading to study termination, were summarized descriptively, by study group and overall; the numbers and percentages (with 95% confidence intervals [CIs]) of study participants reporting these AEs were recorded.

### Statistical Analyses

The study planned to randomize approximately 1541 individuals to achieve 1400 evaluable participants. Assessments for both immunogenicity and safety were considered when determining sample size for the study. Participants who withdrew from the study were not replaced. To ensure the global type I error for the primary objectives was controlled at 5%, each of the primary objectives was tested using a nominal 1-sided alpha of 2.5%. If the primary objective of noninferiority for FLU-D-QIV in terms of GMT was met, the secondary confirmatory objective of noninferiority for RSVPreF3 vaccine in terms of GMC was planned to be tested at an alpha of 2.5%; if this objective was met, the noninferiority of FLU-D-QIV in terms of seroconversion rate was planned to be tested at an alpha of 2.5% for each strain.

For the immunogenicity analyses, the per-protocol set (PPS) was used. The PPS comprised all study participants who received ≥1 dose of the study treatment to which they were randomized and who had postvaccination immunogenicity data (excluding participants with protocol deviations that led to withdrawal from the study). Safety analyses were performed on the exposed set (ES; all participants who received ≥1 dose of the study treatment) or solicited safety set (sSS; all participants who received ≥1 dose of the study intervention [ES], and for whom solicited safety data were available).

### Study Ethics

The study was conducted in accordance with the study protocol and with consensus ethical principles derived from international guidelines, including the Declaration of Helsinki and the Council for International Organizations of Medical Sciences, international ethical guidelines; applicable International Council for Harmonisation Good Clinical Practice guidelines; and all applicable laws and regulations. All study participants provided written (or thumb-printed and witnessed) informed consent prior to the performance of any study-specific procedure.

The study protocol and amendments, the informed consent, and other relevant information were reviewed by national, regional, or investigational center independent ethics committees or institutional review boards. The study was registered with ClinicalTrials.gov (NCT05045144) and the European Union Drug Regulating Authorities Clinical Trials (EudraCT) Database 2021-000357-26.

## RESULTS

### Study Participants

Of the 1586 participants enrolled in the study, 1542 were randomized and 1539 received the study intervention at visit 1 and were included in the ES; 28 participants were withdrawn from the study, primarily because of loss to follow-up (n = 26) (Figure 2). Of the 1539 vaccinated participants (ES), 1537 (99.9%) were included in the sSS and had safety data collected from visit 1 to study end. A total of 1511 participants completed the study. The full analysis set comprised 1522 participants, of whom 1415 were included in the PPS for the immunogenicity analyses.

In study part A, 661 participants received RSV1 (n = 220), RSV2 (n = 223), or RSV3 (n = 218). Of these, 612 were included in the PPS for the immunogenicity analyses.

In study part B, 440 participants were randomized to the RSV + Flu (pooled) group, of whom 438 (99.5%) received the study intervention at visit 1 and were included in the ES. Of these, 398 participants were included in the PPS for the immunogenicity analyses. A total of 440 study participants were randomized to the Flu + P group, all of whom received the study intervention at visit 1 and were included in the ES. Of these, 405 participants were included in the PPS for the immunogenicity analyses.

#### Demographic and Baseline Characteristics

In study part A, demographic and baseline characteristics for the ES were similar for all 3 groups (RSV1, RSV2, and RSV3; Table 1). The mean (standard deviation [SD]) ages of the study participants were 32.0 (9.6), 31.4 (9.4), and 31.9 (9.3) years for the RSV1, RSV2, and RSV3 groups, respectively. Most participants were White and not of Hispanic or Latino ethnicity.

**Table 1.** Summary of Demographic and Baseline Characteristics (Exposed Set)

Characteristic	RSV1 (n = 220)	RSV2 (n = 223)	RSV3 (n = 218)	Pooled RSV (n = 661)	RSV + Flu (Pooled) (n = 438)	Flu + Placebo (n = 440)	Total (n = 1539)
Age, y, mean (SD)	32.0 (9.6)	31.4 (9.4)	31.9 (9.3)	31.8 (9.4)	32.0 (8.9)	32.1 (8.9)	31.9 (9.1)
Age group, No. (%)							
18–32 y	123 (55.9)	123 (55.2)	123 (56.4)	369 (55.8)	243 (55.5)	242 (55.0)	854 (55.5)
33–49 y	97 (44.1)	100 (44.8)	95 (43.6)	292 (44.2)	195 (44.5)	198 (45.0)	685 (44.5)
Country, No. (%)							
Canada	55 (25.0)	61 (27.4)	64 (29.4)	180 (27.2)	124 (28.3)	129 (29.3)	433 (28.1)
Finland	17 (7.7)	13 (5.8)	14 (6.4)	44 (6.7)	29 (6.6)	31 (7.0)	104 (6.8)
Republic of Korea	19 (8.6)	18 (8.1)	18 (8.3)	55 (8.3)	31 (7.1)	31 (7.0)	117 (7.6)
Spain	85 (38.6)	84 (37.7)	80 (36.7)	249 (37.7)	166 (37.9)	161 (36.6)	576 (37.4)
United States	44 (20.0)	47 (21.1)	42 (19.3)	133 (20.1)	88 (20.1)	88 (20.0)	309 (20.1)
Ethnicity, No. (%)							
Hispanic or Latino	10 (4.5)	11 (4.9)	11 (5.0)	32 (4.8)	29 (6.6)	31 (7.0)	92 (6.0)
Not Hispanic or Latino	210 (95.5)	212 (95.1)	207 (95.0)	629 (95.2)	409 (93.4)	409 (93.0)	1447 (94.0)
Race, No. (%)							
American Indian or Alaska Native	2 (.9)	4 (1.8)	3 (1.4)	9 (1.4)	10 (2.3)	7 (1.6)	26 (1.7)
Asian	22 (10.0)	25 (11.2)	21 (9.6)	68 (10.3)	38 (8.7)	43 (9.8)	149 (9.7)
Black or African American	12 (5.5)	3 (1.3)	6 (2.8)	21 (3.2)	17 (3.9)	17 (3.9)	55 (3.6)
Native Hawaiian or other Pacific Islander	0 (0)	0 (0)	0 (0)	0 (0)	1 (.2)	0 (0)	1 (.1)
White	183 (83.2)	187 (83.9)	185 (84.9)	555 (84.0)	369 (84.2)	366 (83.2)	1290 (83.8)
Other	1 (.5)	4 (1.8)	3 (1.4)	8 (1.2)	3 (.7)	7 (1.6)	18 (1.2)
Height, cm, mean (SD)	163.9 (7.1)	163.6 (6.0)	164.2 (6.2)	163.9 (6.4)	164.6 (6.2)	164.6 (6.4)	164. 3 (6.4)
Weight, kg, mean (SD)	68.4 (16.3)	67.9 (16.0)	69.7 (16.2)	68.7 (16.1)	70.1 (15.6)	70.2 (16.1)	69.5 (16.0)
BMI, kg/m^2^, mean (SD)	25.4 (5.6)	25.3 (5.6)	25.8 (5.7)	25.5 (5.6)	25.9 (5.5)	25.9 (5.5)	25.7 (5.6)
Childbearing potential, No. (%)	206 (93.6)	205 (91.9)	200 (91.7)	611 (92.4)	410 (93.6)	415 (94.3)	1436 (93.3)

Abbreviations: BMI, body mass index; Flu, FLU-D-QIV quadrivalent influenza vaccine; RSV, respiratory syncytial virus; RSV1, respiratory syncytial virus prefusion protein F3 (lot 1); RSV2, respiratory syncytial virus prefusion protein F3 (lot 2); RSV3, respiratory syncytial virus prefusion protein F3 (lot 3); SD, standard deviation.

In study part B, demographic and baseline characteristics for the ES were similar for the RSV + Flu (pooled) and Flu + P groups; characteristics were also similar to those for the combined RSV group in study part A. The mean (SD) ages of the study participants were 32.0 (8.9) years in the RSV + Flu (pooled) group and 32.1 (8.9) years in the Flu + P group. Most of the participants were White and not of Hispanic or Latino ethnicity.

### Immunogenicity

#### Study Part A

**Figure 3.**
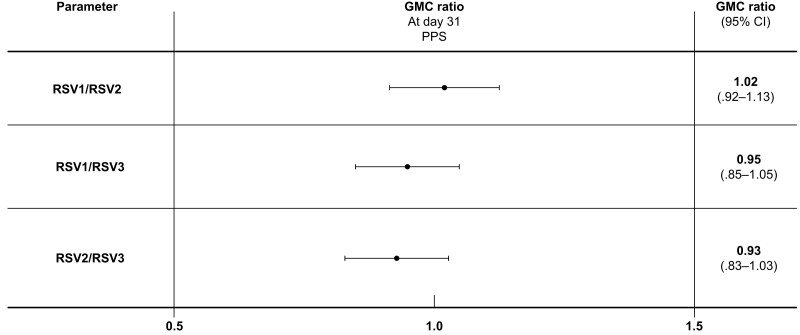
Geometric mean concentration ratios between 2 lots for respiratory syncytial virus prefusion protein F3 IgG antibody concentrations at day 31 postvaccination (per-protocol set). Abbreviations: CI, confidence interval; GMC, geometric mean concentration; IgG, immunoglobulin G; PPS, per-protocol set; RSV1, respiratory syncytial virus prefusion protein F3 (lot 1); RSV2, respiratory syncytial virus prefusion protein F3 (lot 2); RSV3, respiratory syncytial virus prefusion protein F3 (lot 3).

There was a robust immune response at day 31 across each of the 3 RSVPreF3 vaccine lots, and the 2-sided 95% CIs for the 3 pairwise GMC ratios were well within the predefined criteria of 0.67 and 1.50 for each pair of lots (Figure 3). The 95% CIs for adjusted GMC ratios, analyzed to account for prevaccination levels, were also between 0.67 and 1.50. The adjusted GMC ratio (95% CI) was 1.01 (.91–1.12) for RSV1/RSV2, 0.93 (.84–1.03) for RSV1/RSV3, and 0.92 (.83–1.02) for RSV2/RSV3.

#### Study Part B

**Figure 4.**
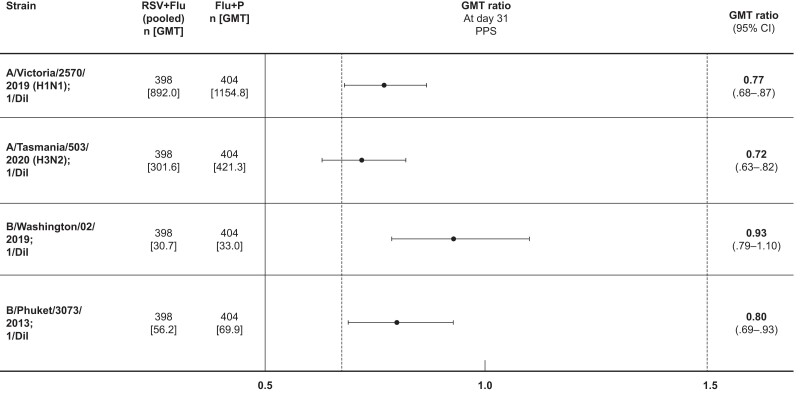
Hemagglutination inhibition antibody geometric mean titer ratios against 4 influenza strains for the respiratory syncytial virus (RSV) + FLU-D-QIV quadrivalent influenza vaccine (Flu) pooled group (RSV1 + Flu, RSV2 + Flu, RSV3 + Flu) divided by the Flu + placebo group at day 31. Dashed lines indicate the predefined noninferiority criteria range of 0.67–1.50. Abbreviations: CI, confidence interval; Dil, dilution; Flu, FLU-D-QIV quadrivalent influenza vaccine; GMT, geometric mean titer; P, placebo; PPS, per-protocol set; RSV, respiratory syncytial virus; RSV1, respiratory syncytial virus prefusion protein F3 (lot 1); RSV2, respiratory syncytial virus prefusion protein F3 (lot 2); RSV3, respiratory syncytial virus prefusion protein F3 (lot 3).

The primary analysis of noninferiority for FLU-D-QIV co-administered with RSVPreF3, compared with FLU-D-QIV administered alone at day 31, was performed on the PPS. The lower limit of the 2-sided 95% CI of the GMT ratio was >0.67 for A/Victoria/2570/2019 (H1N1) (.68), B/Washington/02/2019 (.79), and B/Phuket/3073/2013 (.69), but lower for A/Tasmania/503/2020 (H3N2) (.63) (Figure 4). Thus, the noninferiority criterion was satisfied for 3 influenza strains, but not met for 1 strain. For adjusted GMT ratios, calculated to account for prevaccination levels (ie, individual GMT values were adjusted for baseline values), the lower limit of the 2-sided 95% CI of the GMT ratio was >0.67 for all 4 influenza strains: A/Victoria/2570/2019 (H1N1) (.68), B/Washington/02/2019 (.74), B/Phuket/3073/2013 (.74), and A/Tasmania/503/2020 (H3N2) (.68).

#### Secondary Objectives

**Figure 5.**
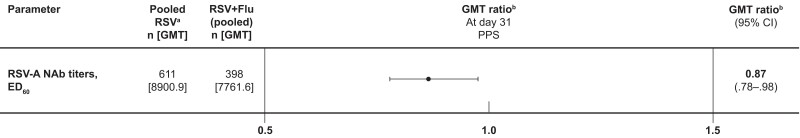
Geometric mean titer ratio for respiratory syncytial virus prefusion protein F3 (RSVPreF3) vaccine co-administered with FLU-D-QIV vaccine versus RSVPreF3 alone, regarding RSV-A neutralizing antibody titers (estimated dilution 60) at day 31 (per-protocol set). ^a^Pooled RSV = pooled data for groups RSV1, RSV2, and RSV3 (ie, for lots 1, 2, and 3 of the RSVPreF3 vaccine). ^b^RSV + Flu (pooled) group divided by pooled RSV group. Abbreviations: CI, confidence interval; ED_60_, estimated dilution 60; Flu, FLU-D-QIV quadrivalent influenza vaccine; GMT, geometric mean titer; NAb, neutralizing antibody; PPS, per-protocol set; RSV, respiratory syncytial virus; RSV1, respiratory syncytial virus prefusion protein F3 (lot 1); RSV2, respiratory syncytial virus prefusion protein F3 (lot 2); RSV3, respiratory syncytial virus prefusion protein F3 (lot 3).

For the immune response for the RSV + Flu (pooled) group versus the RSVPreF3 alone group (pooled RSV) regarding GMTs of RSV-A neutralizing antibodies (NAbs) (estimated dilution 60 [ED_60_]) at day 31 postvaccination, the GMT ratio was 0.87 (95% CI, .78–.98) (Figure 5).

Full data from additional secondary analyses are presented in the Supplementary Materials and Supplementary Table 2. For RSV-A NAbs and RSV-B NAbs, considerable increases in GMTs (above the cut-offs of 18 ED_60_ and 30 ED_60_, respectively) were observed in groups RSV1, RSV2, and RSV3 from day 1 to day 31. For RSV IgG antibody concentrations, a considerable increase in GMCs (above the cut-off of 25 EU/mL) was observed in groups RSV1, RSV2, and RSV3 from day 1 to day 31. For HI GMTs for the 4 constituent influenza strains, robust increases from day 1 to day 31 were noted in immune response in both the RSV + Flu (pooled) and Flu + P groups for all 4 strains. At day 31, seroconversion rates against A/Victoria/2570/2019 (H1N1), A/Tasmania/503/2020 (H3N2), B/Washington/02/2019, and B/Phuket/3073/2013 strains were 72.1%, 42.5%, 25.4%, and 31.4%, respectively, in the RSV + Flu (pooled) group, and 80.4%, 45.9%, 29.5%, and 35.5%, respectively, in the Flu + P group. Corresponding seroprotection rates were 99.7%, 98.0%, 48.0%, and 74.4%, respectively, in the RSV + Flu (pooled) group, and 100%, 98.5%, 48.8%, and 77.2% in the Flu + P group.

### Safety and Reactogenicity

No participants were withdrawn from the study due to AEs or SAEs. For full details of safety and reactogenicity, please refer to the Supplementary Materials.

#### Study Part A

**Figure 6.**
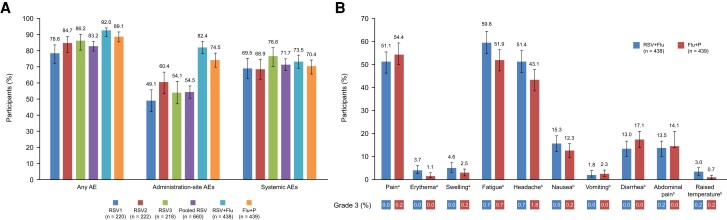
Solicited adverse events (AEs) during the 7-day postvaccination period (solicited safety set). *A*, Any AEs, administration-site AEs, and systemic AEs. *B*, Specific AEs and grade 3 AEs. ^a^Associated with respiratory syncytial virus prefusion protein F3 vaccine, except in the Flu + placebo group, for which the AE incidence was associated with FLU-D-QIV vaccine. ^b^Overall incidence of AEs. Abbreviations: AE, adverse event; Flu, FLU-D-QIV quadrivalent influenza vaccine; P, placebo; RSV, respiratory syncytial virus; RSV1, respiratory syncytial virus prefusion protein F3 (lot 1); RSV2, respiratory syncytial virus prefusion protein F3 (lot 2); RSV3, respiratory syncytial virus prefusion protein F3 (lot 3).

Overall, the proportion of study participants reporting ≥1 AE was similar across all 3 groups. At least 1 solicited AE (occurring within 7 days of vaccination) was reported by 173 (78.6%), 188 (84.7%), and 188 (86.2%) participants in the RSV1, RSV2, and RSV3 groups, respectively (Figure 6); these AEs were administration-site events in 108 (49.1%), 134 (60.4%), and 118 (54.1%) participants. For solicited systemic AEs, the incidence was similar across all 3 groups. In addition, for unsolicited AEs (occurring within 30 days of vaccination), the incidence was similar across all 3 groups. At least 1 unsolicited AE was reported by 60 (27.3%), 60 (26.9%), and 56 (25.7%) participants in the RSV1, RSV2, and RSV3 groups, respectively (Supplementary Table 3).

#### Study Part B

Overall, the proportion of study participants reporting ≥1 AE was similar across both groups. At least 1 solicited AE (occurring within 7 days of vaccination) was reported by 403 (92.0%) and 391 (89.1%) participants in the RSV + Flu (pooled) and Flu + P groups, respectively (Figure 5); these solicited AEs were administration-site events in 361 (82.4%) and 327 (74.5%) participants. For solicited systemic AEs, the incidence was similar across both groups, 322 (73.5%) in the RSV + Flu (pooled) group and 309 (70.4%) in the Flu + P group. For unsolicited AEs (occurring within 30 days of vaccination), the incidence was similar across both groups. At least 1 unsolicited AE was reported by 132 (30.1%) and 113 (25.7%) participants in the RSV + Flu (pooled) and Flu + P groups, respectively (Supplementary Table 3).

#### SAEs

Regarding SAEs from vaccination to study end, 5 (.8%), 4 (.9%), and 4 (.9%) participants reported ≥1 SAE in the pooled RSV, RSV + Flu (pooled), and Flu + P groups, respectively. Only 1 of these SAEs was considered treatment-related by the investigator (1 case of optic neuropathy in the Flu + P group). A total of 6 SAEs (joint ankylosis, suicidal ideation, back pain, fibula fracture, greater trochanteric pain syndrome, and cervical dysplasia) were reported by 5 participants in the pooled RSV group. A total of 5 SAEs (overdose, intervertebral disc protrusion, appendicitis, coronavirus disease 2019 pneumonia, and suicide attempt) were reported by 4 participants in the RSV + Flu (pooled) group. A total of 4 SAEs (ligament rupture, multiple fractures, optic neuropathy, and cholelithiasis) were reported by 4 participants in the Flu + P group. No fatal events were reported.

## DISCUSSION

In this phase 3, randomized study, the lot-to-lot consistency objective was met, as the 2-sided 95% CIs for GMC ratios were within 0.67 and 1.5 for each pair of lots evaluated. There was a robust increase in the immune response when measured in terms of RSV-A and RSV-B NAb titers and in the RSVPreF3 IgG humoral antibody response.

Regarding the effect of the RSVPreF3 vaccine on the FLU-D-QIV vaccine, for 3 constituent influenza strains (A/Victoria/2570/2019 [H1N1], B/Washington/02/2019, and B/Phuket/3073/2013), the predefined noninferiority criterion was met. However, for A/Tasmania/503/2020 (H3N2), the lower limit of the 2-sided 95% CI of the GMT ratio was less than the predefined criterion of success (ie, .67) and the clinical significance of this is unclear. Nevertheless, the noninferiority criterion was met when the adjusted GMT ratio was considered. There was a robust immune response in both the RSV + Flu (pooled) and Flu + P groups, measured in terms of HI titers against the 4 influenza strains.

Considering the effect of FLU-D-QIV on the RSVPreF3 vaccine, as per the hierarchy of confirmatory analyses, no conclusions on the hypothesis for the secondary confirmatory objectives have been presented, as 1 strain did not meet the noninferiority criteria. Nonetheless, there was a robust increase in the immune response generated by RSVPreF3, either alone or co-administered with FLU-D-QIV.

From the safety standpoint, all 3 lots of the RSV vaccine, and the FLU-D-QIV vaccine, were well tolerated. No deaths were reported in the study, and only 1 treatment-related (as considered by the investigator) SAE (optic neuropathy in a participant in the Flu + P group) was reported during the study; the relatedness to FLU-D-QIV is contested by the study sponsor, as there is insufficient evidence to suggest an association. Overall, no safety concerns in this study were identified following co-administration of a single intramuscular dose of RSVPreF3 vaccine with either FLU-D-QIV or placebo in healthy, nonpregnant women aged 18–49 years.

In our study of healthy, nonpregnant women aged 18–49 years, 3 separate lots of RSVPreF3 vaccine demonstrated immunogenic noninferiority, and co-administration of RSVPreF3 with FLU-D-QIV was immunogenically noninferior (for 3 of 4 constituent influenza strains evaluated) to the administration of FLU-D-QIV alone; in addition, clinically acceptable safety and reactogenicity profiles were observed for both vaccines in this study. If the further clinical development of the RSVPreF3 vaccine had not been discontinued, the results of this study would have supported the co-administration of the investigational RSVPreF3 vaccine with FLU-D-QIV at a single clinic visit; indeed, noninferiority of immunogenicity of this co-administration strategy was demonstrated in a study using the adjuvanted vaccine in older adults [20]. This may have afforded pregnant women the option to protect their future offspring from RSV LRTDs, without interfering with protection against influenza virus, thereby reducing the burden of disease from influenza and LRTDs caused by RSV.

## Supplementary Data

Supplementary materials are available at *The Journal of Infectious Diseases* online (http://jid.oxfordjournals.org/). Supplementary materials consist of data provided by the author that are published to benefit the reader. The posted materials are not copyedited. The contents of all supplementary data are the sole responsibility of the authors. Questions or messages regarding errors should be addressed to the author.

## Supplementary Material

jiae342_Supplementary_Data

## Notes

***Acknowledgments.*** The authors would like to thank Dolores Ochoa Mazarro, Diane Carbonneau, Jacob Lee, and all the study investigators. Medical writing support was provided by David Murdoch, a contract writer working on behalf of Apollo, and Alex Coulthard of Apollo, OPEN Health Communications, funded by GSK, in accordance with Good Publication Practice 3 (GPP) guidelines (www.ismpp.org/gpp-2022).

***Author contributions.*** All authors made substantial contributions to the conception or design of the work or to the acquisition, analysis, or interpretation of data for the work. All authors were involved in drafting the work or revising it critically for important intellectual content. All authors provided final approval of the version to be published. All authors provided agreement to be accountable for all aspects of the work in ensuring that questions related to the accuracy or integrity of any part of the work are appropriately investigated and resolved.

***Data availability.*** The study report, including the protocol, is available on the GSK Clinical Study Register (https://www.gsk-studyregister.com). Anonymized individual participant data and study documents can be requested for further research from www.clinicalstudydatarequest.com (study ID 214709).

***Financial support*.** This study was sponsored by GSK.
