# Supplementary material for: Phase 3 Study Assessing Lot-to-Lot Consistency of Respiratory Syncytial Virus Prefusion Protein F3 Vaccine and Its Immune Response, Safety, and Reactogenicity When Co-administered With Quadrivalent Influenza Vaccine
Source: J Infect Dis. 2024 Jul 5;231(1):e144–53. doi: 10.1093/infdis/jiae342 (PMC11793055; doi:10.1093/infdis/jiae342)
Supplement: jiae342_Supplementary_Data [file jiae342_supplementary_data.docx]

**Supplementary Materials**

**Study Design and Participants**

The study was conducted at 36 centers in 5 countries: Canada (n = 11), Finland (9), Spain (8), USA (5), and Republic of Korea (3).

**Exclusion Criteria**

- A history of any reaction or hypersensitivity likely to be exacerbated by any component of the study interventions
- Any confirmed or suspected immunosuppressive or immunodeficient condition, based on medical history and physical examination (no laboratory testing required)
- A current autoimmune disorder (based on medical history and physical examination), for which the participant had received immune-modifying therapy within the prior 6 months
- Hypersensitivity to latex
- Acute or chronic, clinically significant abnormalities, or poorly controlled pre-existent comorbidities, or any other clinical conditions (as determined by physical examination or medical history) that, in the investigator’s opinion, might pose additional risk to an individual because of study participation
- Significant or uncontrolled psychiatric illness
- Recurrent history of, or uncontrolled, neurologic disorders or seizures
- Documented human immunodeficiency virus infection
- Body mass index >40 kg/m^2^
- Any hematologic parameter and/or biochemical laboratory abnormality considered clinically significant by an investigator
- Use of any investigational or non-registered product (drug, vaccine, or medical device), other than a study intervention, during the period starting 30 days before study intervention (day –29 to day 1), or planned use during the study period
- Administration of long-acting immune-modifying drugs (eg, infliximab) at any time during the study
- Administration of immunoglobulins (Igs) and/or any blood products or plasma derivatives in the 3 months prior to the study, or planned administration during the study period
- Chronic administration (defined as >14 days in total) of immunosuppressants or other immune-modifying drugs during the period starting 3 months prior to the first study vaccine dose (for corticosteroids, this was a mean prednisone dosage of 5 mg/day, or equivalent; inhaled and topical steroids were allowed)
- Planned administration or administration of a vaccine not foreseen by the study protocol, within the period starting 30 days before and ending 30 days after a study vaccination dose
- Administration of a seasonal influenza vaccine during the 6 months prior to study entry
- Prior experimental vaccination against respiratory syncytial virus (RSV)
- Concurrent participation in another clinical study (at any time during the study period), in which a study participant has been or will be exposed to an investigational or non-investigational vaccine or product (drug or medical device)
- Pregnant or lactating females
- Females planning to become pregnant or planning to discontinue contraceptive precautions
- Alcoholism or substance use disorder within the prior 24 months
- Any study personnel or their immediate dependents, family, or household members

**Randomization**

Randomization was performed using an automated, internet-based system (Source Data Base for Internet Randomization), and a randomization algorithm was used with a minimization procedure accounting for age at the time of vaccination (18–32 versus 33–49 years) and study center. Minimization factors had equal weight in the algorithm. Following allocation of a participant identification number, the randomization system determined the study group and provided the study intervention number to be used for the first dose.

**Blinding**

Data were collected in an observer-blind manner to evaluate the lot-to-lot consistency of RSV prefusion protein F3 (RSVPreF3) vaccine, and in a single-blind manner to evaluate the immune response, safety, and reactogenicity of RSVPreF3 co-administered with FLU-D-QIV. Study interventions were prepared and administered by qualified study personnel who did not participate in data collection, evaluation, or review, or the entry of any study endpoint (ie, immunogenicity, safety, reactogenicity).

In the observer-blind design (Part A), for the lot-to-lot consistency groups (RSV1, RSV2, and RSV3), study participants and the investigators were blinded to the lot received; however, the participants and investigators were aware that participants were not part of the co-administration study groups. In the single-blind design (Part B), for the co-administration groups (RSV+Flu or Flu+P), the study participants and investigators were aware that the participants were part of the co-administration study groups; however, the participants were blinded to the vaccine administration activities. In both study parts, participants received either RSVPreF3 or FLU-D-QIV in the left arm and FLU-D-QIV or placebo in the right arm. In study Part A, the FLU-D-QIV dose at day 31 was administered in the non-dominant arm (**Supplementary** **Table 1**). Both blinding strategies were applied from visit 1 (day 1) to study end (day 181).

**Secondary Descriptive Objectives**

Secondary, descriptive immunogenicity objectives were to evaluate the humoral immune responses to RSVPreF3 alone, and when co-administered with FLU-D-QIV, regarding GMTs of RSV-A and RSV-B NAbs, and geometric mean concentrations (GMCs) of RSVPreF3 IgG, at days 1 and 31; the humoral immune response to the FLU-D-QIV vaccine alone, or when co-administered with RSVPreF3, regarding HI GMTs for 4 constituent influenza strains at days 1 and 31; the seroprotection and seroconversion rates for the FLU-D-QIV vaccine alone, and when co-administered with RSVPreF3; and the humoral immune responses to the 3 separate lots of RSVPreF3, based on GMTs of RSV-A and RSV-B NAbs, and GMCs of RSVPre3 IgG antibodies, at days 1 and 31.

**Immunogenicity Assays**

To date, no generally accepted immunologic correlate of protection has been demonstrated for the antigens used in the RSVPreF3 vaccine. In addition, although there is no accepted correlate of immunity against influenza, the protective roles of antibodies against hemagglutinin and, to a lesser extent, neuraminidase, are well established and have been demonstrated both in experimentally infected animals and in humans [1]. Thus, the induction of hemagglutinin-specific antibodies was used as a marker of potential vaccine efficacy, and the serum HI assay was used to demonstrate this humoral response. HI antibody titers ≥1:40 have been associated with protection from influenza illness in ≥50% of participants in challenge studies [2], and correlate with vaccine effectiveness [3].

RSVPreF3 enzyme-linked immunosorbent assay (ELISA)

The RSVPreF3 IgG ELISA is based on an indirect ELISA allowing the detection and the quantification of total IgG antibodies directed against RSVPreF3 in human serum samples. The principle of this assay is as follows: RSVPreF3 antigen is adsorbed onto a 96-well polystyrene microplate. After a washing and a blocking step, dilutions of serum samples, controls and standards are added to the coated microplate. A reference standard curve is prepared using a pool of commercial human serum containing anti-RSV antibodies. After incubation, the microplate is washed to remove unbound primary antibodies. Bound IgG is detected by the addition of a secondary anti-human antibody conjugated to HRP. Bound antibodies are quantified by the addition of the HRP substrate, tetramethylbenzidine and hydrogen peroxide, whereby a colored product develops proportionally to the amount of anti-RSVPreF3 IgG antibodies present in the serum sample. The optical density of each sample dilution is then interpolated on the reference standard. The corresponding antibody concentration, corrected for the dilution factor, is expressed in arbitrary ELISA Laboratory Units per milliliter (EU/mL).

RSV-A/B neutralization assay

The serum neutralization assay is a functional assay that measures the ability of serum antibodies to neutralize RSV entry and replication in a host cell line. First, virus neutralization is performed by incubating a fixed amount of RSV-A strain (Long) or RSV-B strain (B18537) with serial dilutions of the test serum. Then, the serum/virus mixture is transferred onto a monolayer of Vero cells (African Green Monkey, kidney, Cercopitheus aethiops, ATCC CCL-81) and incubated for two days to allow infection and the formation of plaques by non-neutralized viruses. Following the fixation period, RSV-infected cells are detected using a primary antibody directed against RSV (anti-RSV IgG) and a secondary antibody conjugated with horse-radish peroxidase (HRP), allowing the visualization of plaques after coloration with TrueBlueTM peroxidase substrate. Viral plaques are counted using an automated microscope coupled to an image analyzer (Scanlab system with Axiovision software). For each serum dilution, a ratio, expressed as a percentage, is calculated between the number of plaques at that dilution and the number of plaques in the virus control wells (no serum added). The serum neutralizing titer is expressed in ED60 (Estimated Dilution 60) and corresponds to the inverse of the interpolated serum dilution that yields a 60% reduction in the number of plaques compared to the virus control wells as described by others [4, 5].

FLU Hemagglutination-inhibition (HI) assay

Hemagglutination inhibition (HI) antibody titres were determined using the method derived from the WHO Manual on Animal Influenza Diagnosis and Surveillance, WHO/CDS/CSR/NCS/2002.5.

Measurements are conducted on thawed frozen serum samples with a standardized and comprehensively validated micro-method. Briefly, serum samples are treated with receptor destroying enzyme (RDE) overnight, diluted to 1:10, and serial diluted 2- fold in triplicate from 1:10 to 1:10240. After addition of an equal volume of standardized virus (4 HA / 25 μL), neutralization is performed for 1 hour at room temperature, followed by addition of the RBCs. After 30 minutes, plates are tilted and the titer is the reciprocal of the last dilution that fully inhibits hemagglutination as compared to a RBC control well. Each sera sample will be tested in triplicate within the same assay. The three titer results will be reported as will the geometric mean titer (GMT) for the triplicate.

**Statistical Analyses**

The primary objective of lot-to-lot consistency was evaluated on RSVPreF3 immunogenicity data, as measured by RSVPreF3 IgG enzyme-linked immunosorbent assay (ELISA) concentration at day 31. Lot-to-lot consistency was demonstrated only if two-sided 95% confidence intervals (CIs) for the 3 pair-wise GMC ratios for RSVPreF3 IgG ELISA at day 31 (30 days post-vaccination) were within the 0.67–1.50 range. With the assumptions of a standard deviation (SD) for the log_10_ transformed RSVPreF3 IgG ELISA concentration of 0.35, true GMC ratio of one between two lots, type I error of 0.025, and 10% nonevaluable rate, 220 enrollees per lot provided >99% global power to conclude lot-to-lot consistency.

The potential interference of RSVPreF3 vaccine with influenza immune responses was evaluated using FLU-D-QIV HI antibody GMT ratios against 4 influenza strains, for the RSV+Flu pooled (RSV1+Flu, RSV2+Flu, RSV3+Flu) and Flu+P groups at day 31. The hypothesis was that co-administration of RSVPreF3 with FLU-D-QIV would be noninferior to FLU-D-QIV alone, regarding GMT ratio for the immune response of influenza antigens in non-pregnant women at day 31 post-vaccination. The criterion for noninferiority for the A/Victoria/2570/2019 (H1N1) IVR-215, A/Tasmania/503/2020 (H3N2) IVR-221, B/Washington/02/2019, and B/Phuket/3073/2013 strains was that the lower limit of the 95% CI for the GMT ratio (RSV+Flu pooled group divided by Flu+P group) was greater than 0.67 at day 31 post-vaccination. The statistical power would be ≥95% for a sample size of 400 participants per group, with a SD of 0.6 for the log_10_ transformation, and a noninferiority margin of 0.67, between the RSV+Flu pooled and Flu+P groups.

To control the global type I error, a hierarchical procedure was used for assessment of the confirmatory objectives. For instance, the secondary objective to demonstrate the non-inferiority of RSV-A neutralizing antibodies using GMT ratios was to be assessed only if the primary objective assessing the non-inferiority of GMT ratio for FLU-D-QIV strains was met. As per the hierarchical procedure, the secondary objective assessing the non-inferiority based on seroconversion rate difference of FLU-D-QIV was to be assessed only if the non-inferiority of RSV-A neutralizing antibodies using GMT ratios was met.

For the primary safety objectives, sample sizes of 660 participants (3 lots pooled), and 440 participants per group in the co-administration group (RSV+Flu [pooled]), at the first vaccination were planned to provide reasonable confidence to evaluate adverse event (AE) rates. If an AE was not observed in an RSV treatment group (RSV1, RSV2, or RSV3), a sample size of 660 participants for pooled RSV lots could provide ≥95% confidence to rule out an AE incidence >0.56%. A sample size of 440 vaccinated participants in the RSV+Flu (pooled) group would provide ≥95% confidence to rule out an AE incidence rate >0.83%, and would provide a probability of 98.8% of observing ≥1 AE if the true AE rate was 1%.

For the hypothesis that the immune response to co-administration of RSVPreF3 + FLU-D-QIV was noninferior to that when RSV PreF3 was administered alone (pooled RSV1, RSV2, and RSV3 group), regarding GMT ratio for RSV-A NAb titer at day 31 post-vaccination, the criterion for noninferiority was that the lower limit of the 95% CI for the GMT ratio (RSV+Flu group divided by the pooled RSV group) was greater than the pre-defined clinical limit of 0.67 at day 31 post-vaccination. Assuming a SD of 0.4 for the log_10_ transformed RSV-A NAb GMT, and a GMT ratio of 1.0, there would be a ≥99% chance that the lower bound of the 95% CI for the GMT ratio for RSV-A NAb titer for the RSV+Flu group versus the pooled RSV groups would be >0.67.

The potential interference of RSVPreF3 vaccine with influenza immune responses was evaluated by comparing the day 31 seroconversion rate regarding HI antibody titers against 4 constituent influenza strains (Flu+P group versus RSV+Flu group). The criterion for noninferiority with respect to the seroconversion rate difference for antibody titers against the 4 influenza strains was that the upper limit of the 95% CI for the seroconversion rate difference (Flu+P minus RSV+Flu) was less than the pre-defined clinical limit of 10% at day 31 post-vaccination. Assuming a seroconversion rate of 65% for the reference group, a significance level 0.025, and a noninferiority margin of 10%, 400 evaluable participants (10% nonevaluable rate) would provide ≥80% power to conclude noninferiority for each influenza strain.

Pre-vaccination levels were considered for assessment in exploratory analyses using the ANCOVA model with pre-vaccination titers/ concentrations as one of the covariates. No type 1 error adjustments were made as these analyses were descriptive.

**Results for Additional Secondary Objectives**

In secondary analyses of RSV-A NAbs, a considerable increase in GMTs (above the cut-off of 18 estimated dilution 60 [ED60]) was observed for group RSV1 (GMT 8545.5; 11.6-fold increase), group RSV2 (GMT 8760.3; 12.3-fold increase), and group RSV3 (GMT 9409.2; 13.8-fold increase) from day 1 to day 31. In addition, there was a considerable increase in GMTs (ED60) for both the pooled RSV group (GMT 8900.9; 12.5-fold increase) and the RSV+Flu group (GMT 7761.6; 11.0-fold increase) from day 1 to day 31.

In secondary analyses of RSV-B NAbs, a considerable increase in GMTs (above the cut-off of 30 ED60) was observed for group RSV1 (GMT 10 457.0; 11.6-fold increase), group RSV2 (GMT 11 107.2; 10.9-fold increase), and group RSV3 (GMT 11 543.3; 11.8-fold increase) from day 1 to day 31. In addition, there was a considerable increase in GMTs (above the cut-off of 30 ED60) for both the pooled RSV group (GMT 11 030.5; 11.4-fold increase) and the RSV+Flu group (GMT 9162.3; 9.9-fold increase) from day 1 to day 31.

In secondary analyses of RSV IgG antibody concentrations, a considerable increase in GMCs (above the cut-off of 25 EU/mL) was observed for group RSV1 (GMC 102 811.0; 18.2-fold increase), group RSV2 (GMC 100 635.8; 18.3-fold increase), and group RSV3 (GMC 108 709.3; 20.7-fold increase) from day 1 to day 31. In addition, there was a considerable increase in GMCs (EU/mL) for both the pooled RSV group (GMC 104 025.1; 19.0-fold increase) and the RSV+Flu group (GMC 83 937.0; 14.6-fold increase) from day 1 to day 31.

In secondary analyses of HI GMTs for the 4 constituent influenza strains, robust increases from day 1 to day 31 were noted in immune response in both the RSV+Flu (pooled) and Flu+P groups for all 4 strains. For A/Victoria/2570/2019 (H1N1), HI titers increased to 892.0 (9.7-fold increase) in the RSV+Flu (pooled) group and to 1154.8 (14.2-fold increase) in the Flu+P group. Corresponding increases in HI titers against A/Tasmania/503/2020 (H3N2) were to 301.6 (3.3-fold) and 421.3 (3.8-fold increase), and increases of similar magnitude were noted in both the RSV+Flu (pooled) and Flu+P groups for the other two influenza strains.

At day 31, seroconversion rates against A/Victoria/2570/2019 (H1N1), A/Tasmania/503/2020 (H3N2), B/Washington/02/2019, and B/Phuket/3073/2013 strains were 72.1%, 42.5%, 25.4%, and 31.4%, respectively, in the RSV+Flu (pooled) group, and 80.4%, 45.9%, 29.5%, and 35.5%, respectively, in the Flu+P group (**Supplementary Table 2**). At day 31, seroprotection rates against A/Victoria/2570/2019 (H1N1), A/Tasmania/503/2020 (H3N2), B/Washington/02/2019, and B/Phuket/3073/2013 strains were 99.7%, 98.0%, 48.0%, and 74.4%, respectively, in the RSV+Flu (pooled) group, and 100%, 98.5%, 48.8%, and 77.2%, respectively, in the Flu+P group.

**Safety and Reactogenicity**

*Study Part A*

At least one solicited AE (occurring within 7 days of vaccination) was reported by 173 (78.6%), 188 (84.7%), and 188 (86.2%) participants. These AEs were administration-site events in 108 (49.1%), 134 (60.4%), and 118 (54.1%) participants. Pain was the most frequently reported solicited administration-site event, reported by 108 (49.1%), 131 (59.0%), and 115 (52.8%) participants, and lasting a mean of 2.5, 2.4, and 2.4 days. Grade 3 pain was reported by one (0.5%) participant in each of the RSV1 and RSV2 groups, and by none in the RSV3 group. The incidence of erythema and swelling (4.1–5.0%) was similar across the 3 groups.

For solicited systemic AEs, the incidence was similar across all 3 groups. Fatigue and headache were the most frequently reported solicited systemic AEs: fatigue was reported by 110 (50.0%), 112 (50.5%), and 121 (55.5%) participants and lasted a mean of 2.2, 2.1, and 2.0 days; and headache was reported by 104 (47.3%), 105 (47.3%), and 105 (48.2%) participants and lasted a mean of 1.9, 1.9, and 2.0 days. Grade 3 fatigue was reported by 4 (1.8%), 0 (0%), and 1 (0.5%) participant, and grade 3 headache was reported by 3 (1.4%), 2 (0.9%), and 1 (0.5%) participant.

For unsolicited AEs (occurring within 30 days of vaccination), the incidence was similar across the 3 groups. At least one unsolicited AE was reported by 60 (27.3%), 60 (26.9%), and 56 (25.7%) participants. Grade 3 unsolicited AEs, by preferred term, were headache (treatment-related) and suicidal ideation, reported by one participant each in group RSV1. In addition, one participant in group RSV3 reported grade 3 pain.

*Study Part B*

At least one solicited AE (occurring within 7 days of vaccination) was reported by 403 (92.0%) and 391 (89.1%) participants in the RSV+Flu and Flu+P groups, respectively. These AEs were administration-site events in 361 (82.4%) and 327 (74.5%) participants. Pain was the most frequently reported solicited administration-site event: reported by 224 (51.1%) participants and lasting a mean of 2.2 days in the RSV+Flu (pooled) group; none of these AEs was grade 3. For solicited systemic AEs, the incidence was similar across the groups. Fatigue and headache were the most frequently reported events: fatigue was reported by 262 (59.8%) and 228 (51.9%) participants and lasted a mean of 2.3 and 2.1 days; and headache was reported by 225 (51.4%) and 189 (43.1%) participants and lasted a mean of 1.9 and 1.8 days. Grade 3 fatigue was reported by 3 (0.7%) and 3 (0.7%) participants, and grade 3 headache was reported by 3 (0.7%) and 8 (1.8%) participants.

For unsolicited AEs (occurring within 30 days of vaccination), the incidence was similar across the groups. At least one unsolicited AE was reported by 132 (30.1%) and 113 (25.7%) participants. The most frequently reported unsolicited AEs, by system organ class (SOC), were infections and infestations (45 [10.3%] and 37 [8.4%] participants), followed by general disorders and administration-site conditions (34 [7.8%] and 26 [5.9%] participants), and nervous system disorders (21 [4.8%] and 22 [5%] participants). Treatment-related unsolicited AEs were reported by 55 (12.6%) and 43 (9.8%) participants. The most frequently reported treatment-related unsolicited AEs, by SOC, were general disorders and administration-site conditions (28 [6.4%] and 17 [3.9%] participants). Grade 3 unsolicited AEs were reported by 7 (1.6%) and 2 (0.5%) participants. Treatment-related grade 3 unsolicited AEs were dizziness and migraine, each reported by one (0.2%) participant in the RSV+Flu (pooled) group, and pneumonia and dyspnea, each reported by one (0.2%) participant in the Flu+P group.

*Other Safety Considerations*

A total of 6 pregnancies were reported by the data lock point, 3 in the pooled RSV group and 3 in the Flu+P group. A total of 2 pregnancies in the pooled RSV group were completed to term, and the outcome was live birth with no congenital anomalies; and one pregnancy in the Flu+P group was electively terminated at 6 weeks of gestational age. The other 3 pregnancies were ongoing at the time of data lock.

**Supplementary Table 1.** Study Intervention Administered

| **Day 1 Vaccination** | | | | | |
| --- | --- | --- | --- | --- | --- |
| **Interventional Group** | **n** | **Intervention** | **Laterality** | **Intervention** | **Laterality** |
| RSV1 | 220 | RSVPreF3 120 μg (Lot 1) | Left arm |  |  |
| RSV2 | 220 | RSVPreF3 120 μg (Lot 2) | Left arm |  |  |
| RSV3 | 220 | RSVPreF3 120 μg (Lot 3) | Left arm |  |  |
| RSV1+Flu | 147 | RSVPreF3 120 μg (Lot 1) | Left arm | FLU-D-QIV | Right arm |
| RSV2+Flu | 147 | RSVPreF3 120 μg (Lot 2) | Left arm | FLU-D-QIV | Right arm |
| RSV3+Flu | 147 | RSVPreF3 120 μg (Lot 3) | Left arm | FLU-D-QIV | Right arm |
| Flu+P | 440 | FLU-D-QIV | Left arm | Placebo | Right arm |
| **Day 31 Standard of Care Vaccination^a^** | | | | | |
| **Interventional Group** | **n** | **Intervention** | | **Laterality** | |
| RSV1 | 220 | FLU-D-QIV | | Non-dominant arm | |
| RSV2 | 220 | FLU-D-QIV | | Non-dominant arm | |
| RSV3 | 220 | FLU-D-QIV | | Non-dominant arm | |

^a^Visit 2 Flu-D-QIV vaccinations were not part of the experimental design and served as an optional vaccination for participants to provide standard of care.

Abbreviations: Flu, FLU-D-QIV quadrivalent influenza vaccine; P, placebo; RSV, respiratory syncytial virus; RSVPreF3, RSV prefusion protein F3; RSV1, RSVPreF3 (lot 1); RSV2, RSVPreF3 (lot 2); RSV3, RSVPreF3 (lot 3).

**Supplementary Table 2.** SCR Difference Between FLU-D-QIV Vaccine Alone and FLU-D-QIV Co-administered With RSVPreF3 Vaccine for the 4 Influenza Strains at Day 31 Post-vaccination (Per-protocol Set)

| **Strain** | **SCR** | **RSV+Flu (pooled)** | **Flu+P** | **Difference in SCR (Flu+P – RSV+Flu [pooled])** |
| --- | --- | --- | --- | --- |
| A/Victoria/2570/2019 (H1N1); 1/Dil | N  n  SCR (%)  95% CI | 398  287  72.1  (67.4, 76.5) | 403  324  80.4  (76.2, 84.2) | 8.29  (2.40, 14.16) |
| A/Tasmania/503/2020 (H3N2); 1/Dil | N  n  SCR (%)  95% CI | 398  169  42.5  (37.6, 47.5) | 403  185  45.9  (41.0, 50.9) | 3.44  (–3.44, 10.29) |
| B/Washington/02/2019; 1/Dil | N  n  SCR (%)  95% CI | 398  101  25.4  (21.2, 30.0) | 403  119  29.5  (25.1, 34.2) | 4.15  (–2.04, 10.32) |
| B/Phuket/3073/2013; 1/Dil | N  n  SCR (%)  95% CI | 398  125  31.4  (26.9, 36.2) | 403  143  35.5  (30.8, 40.4) | 4.08  (–2.46, 10.58) |

Abbreviations: CI, confidence interval; Dil, dilution; Flu, FLU-D-QIV quadrivalent influenza vaccine; P, placebo; RSV, respiratory syncytial virus; RSVPreF3, RSV prefusion protein F3; SCR, seroconversion rate.

**Supplementary Table 3.** Summary of Unsolicited AEs^a^ Within 30 Days Post-vaccination (Exposed Set)

| **AE, n (%)** | **RSV1**  **(n = 220)** | **RSV2**  **(n = 223)** | **RSV3**  **(n = 218)** | **Pooled RSV**  **(n = 661)** | **RSV+Flu**  **(n = 438)** | **Flu+P**  **(n = 440)** |
| --- | --- | --- | --- | --- | --- | --- |
| Any AE | 60 (27.3) | 60 (26.9) | 56 (25.7) | 176 (26.6) | 132 (30.1) | 113 (25.7) |
| Nasopharyngitis | 4 (1.8) | 4 (1.8) | 2 (0.9) | 10 (1.5) | 13 (3.0) | 15 (3.4) |
| Upper respiratory tract infection | 4 (1.8) | 0 (0.0) | 3 (1.4) | 7 (1.1) | 8 (1.8) | 2 (0.5) |
| COVID-19 | 0 (0.0) | 2 (0.9) | 4 (1.8) | 6 (0.9) | 7 (1.6) | 2 (0.5) |
| Bronchitis | 0 (0.0) | 0 (0.0) | 3 (1.4) | 3 (0.5) | 1 (0.2) | 3 (0.7) |
| Influenza | 3 (1.4) | 0 (0.0) | 1 (0.5) | 4 (0.6) | 2 (0.5) | 0 (0.0) |
| Axillary pain | 4 (1.8) | 5 (2.2) | 4 (1.8) | 13 (2.0) | 6 (1.4) | 2 (0.5) |
| Fatigue | 2 (0.9) | 1 (0.4) | 2 (0.9) | 5 (0.8) | 6 (1.4) | 5 (1.1) |
| Swelling | 5 (2.3) | 1 (0.4) | 1 (0.5) | 7 (1.1) | 3 (0.7) | 1 (0.2) |
| Injection site pain | 4 (1.8) | 1 (0.4) | 0 (0.0) | 5 (0.8) | 1 (0.2) | 5 (1.1) |
| Injection site lymphadenopathy | 0 (0.0) | 3 (1.3) | 2 (0.9) | 5 (0.8) | 0 (0.0) | 2 (0.5) |
| Chills | 0 (0.0) | 2 (0.9) | 0 (0.0) | 2 (0.3) | 6 (1.4) | 1 (0.2) |
| Headache | 13 (5.9) | 7 (3.1) | 6 (2.8) | 26 (3.9) | 17 (3.9) | 18 (4.1) |
| Oropharyngeal pain | 3 (1.4) | 3 (1.3) | 5 (2.3) | 11 (1.7) | 6 (1.4) | 6 (1.4) |
| Cough | 2 (0.9) | 2 (0.9) | 1 (0.5) | 5 (0.8) | 8 (1.8) | 6 (1.4) |
| Rhinorrhea | 1 (0.5) | 2 (0.9) | 4 (1.8) | 7 (1.1) | 3 (0.7) | 5 (1.1) |
| Nasal congestion | 0 (0.0) | 3 (1.3) | 2 (0.9) | 5 (0.8) | 6 (1.4) | 4 (0.9) |
| Myalgia | 2 (0.9) | 4 (1.8) | 1 (0.5) | 7 (1.1) | 4 (0.9) | 5 (1.1) |
| Arthralgia | 3 (1.4) | 0 (0.0) | 1 (0.5) | 4 (0.6) | 3 (0.7) | 3 (0.7) |
| Diarrhea | 1 (0.5) | 3 (1.3) | 0 (0.0) | 4 (0.6) | 5 (1.1) | 2 (0.5) |
| Lymphadenopathy | 6 (2.7) | 7 (3.1) | 5 (2.3) | 18 (2.7) | 6 (1.4) | 4 (0.9) |

^a^AEs listed according to preferred term; only AEs with an incidence of >1% in any one group are listed.

Abbreviations: AE, adverse event; Flu, FLU-D-QIV quadrivalent influenza vaccine; P, placebo; RSV, respiratory syncytial virus; RSVPreF3, RSV prefusion protein F3; RSV1, RSVPreF3 (lot 1); RSV2, RSVPreF3 (lot 2); RSV3, RSVPreF3 (lot 3).

**References**

1. Brydak LB, Machala M. Humoral immune response to influenza vaccination in patients from high risk groups. Drugs **2000**; 60:35-53.

2. Hannoun C, Megas F, Piercy J. Immunogenicity and protective efficacy of influenza vaccination. Virus Res **2004**; 103:133-8.

3. Beyer WE, Palache AM, Baljet M, Masurel N. Antibody induction by influenza vaccines in the elderly: a review of the literature. Vaccine **1989**; 7:385-94.

4. Barbas CF, 3rd, Crowe JE, Jr., Cababa D, et al. Human monoclonal Fab fragments derived from a combinatorial library bind to respiratory syncytial virus F glycoprotein and neutralize infectivity. Proc Natl Acad Sci U S A **1992**; 89:10164-8.

5. Bates JT, Keefer CJ, Slaughter JC, Kulp DW, Schief WR, Crowe JE, Jr. Escape from neutralization by the respiratory syncytial virus-specific neutralizing monoclonal antibody palivizumab is driven by changes in on-rate of binding to the fusion protein. Virology **2014**; 454-455:139-44.
